# Supplementary material for: Automated microinjection for zebrafish xenograft models
Source: NPJ Biomed Innov. 2025 Apr 23;2:13. doi: 10.1038/s44385-025-00016-y (PMC13055049; doi:10.1038/s44385-025-00016-y)
Supplement: Supplementary file 1 — Supplementary information [file 44385_2025_16_MOESM1_ESM.pdf]

Supplementary information

Automated microinjection for zebrafish xenograft models

Ding et al.

Supplementary figures 1-2 and corresponding figure legends

Supplementary movie captions 1-15

Supplementary Figure 1

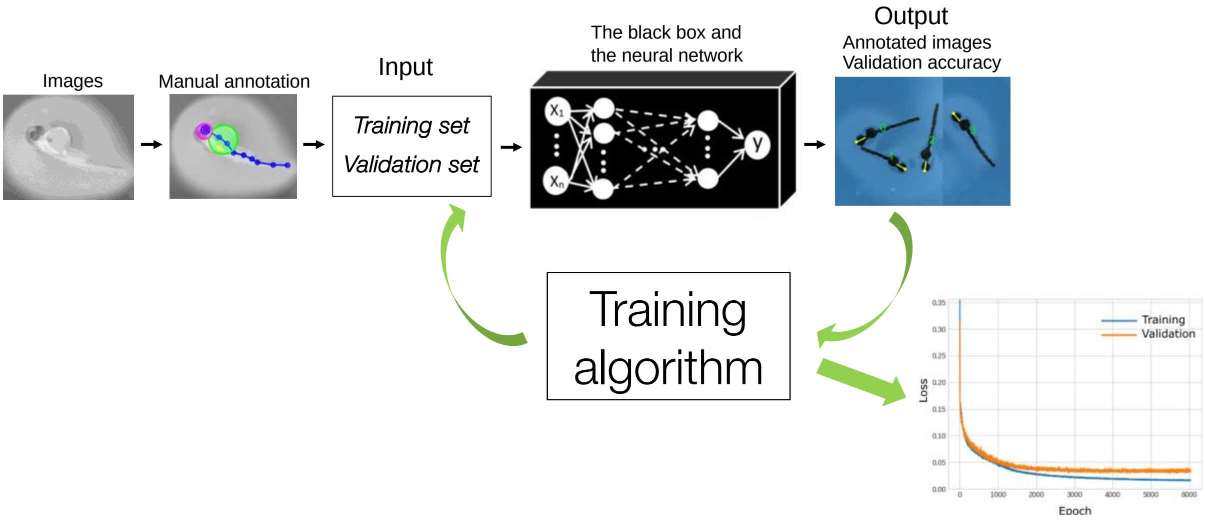

Supplementary Figure 1. Image annotation and deep learning workflow for zebrafish larvae recognition. Images of zebrafish larvae acquired from the microinjection robot are manually annotated and divided into training and validation datasets. The training dataset trains a neural network to recognize zebrafish larvae, while the validation dataset evaluates its performance. The graph illustrates training and validation curves, providing insights into the neural network's learning progression and accuracy.

Supplementary Figure 2

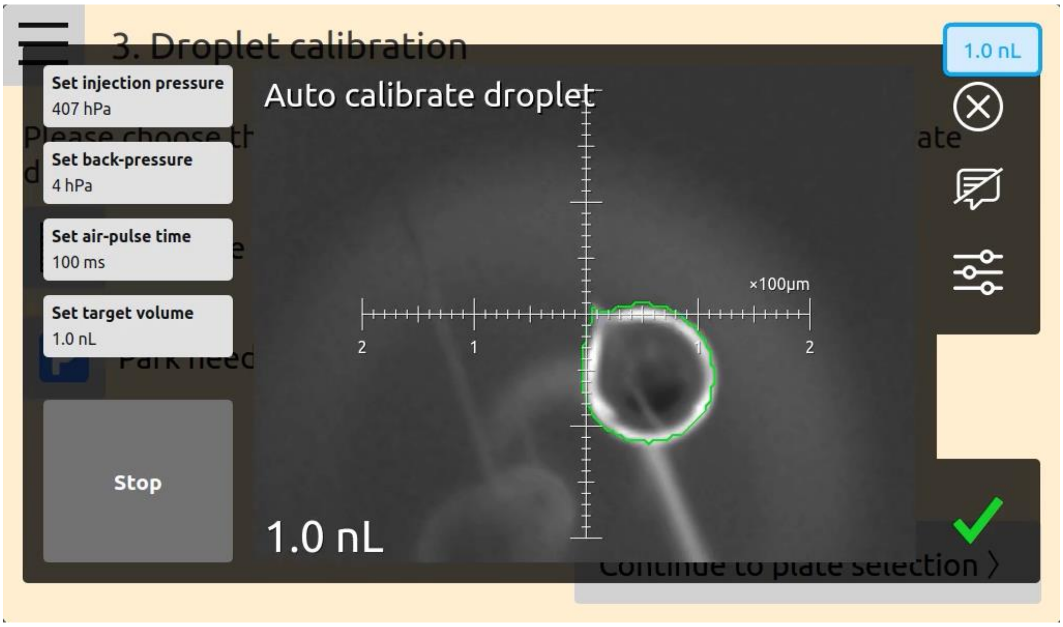

Supplementary Figure 2. Droplet size measurement. A green circle highlights the droplet produced. The droplet volume is automatically calculated and displayed. The scale bar is provided for visual reference and inspection.

Supplementary Movie 1. Needle rotation. This video demonstrates the automated rotation of the needle around the head of the robot for precise positioning.

Supplementary Movie 2. High numerical aperture (NA) illumination. The larva is illuminated under high NA conditions, focusing on the surface features of the larva.

Supplementary Movie 3. Low numerical aperture (NA) illumination. The larva is illuminated under low NA conditions, highlighting deeper structures within the larva.

Supplementary Movie 4. Injection settings. This video outlines the process of choosing injection settings, including selecting the developmental stage, injection site, injection location, and macros.

Supplementary Movie 5. Needle calibration. The needle tip is calibrated by manually aligning it to the center of the circle displayed on the screen using the robot's adjustment screws and focusing on the needle tip. The z-direction calibration is performed automatically, and the positions of each needle rotation are displayed.

Supplementary Movie 6. Droplet calibration. A 6-well plate is placed in the left plate holder, with one well filled with mineral oil and another with water. After selecting the well positions, the "Make Droplet" button is used to produce droplets based on current pressure and air-pulse time settings, which can also be adjusted manually. The droplet size is automatically detected. The "Clean" button applies maximum injection pressure to unblock the needle, and the "Auto Improve" button automatically adjusts settings to achieve the target droplet volume.

Supplementary Movie 7. Puncture detection. The anchor points of larvae around the needle tip are automatically labeled. As the needle punctures the skin of the larvae, the shift in anchor points indicates successful penetration.

Supplementary Movie 8. Automatic injection of phenol red into the duct of Cuvier (DoC). The robot automatically scans the agarose gel plate to locate a larva. Once detected, the needle tip approaches the DoC, adjusts its position, and executes a predetermined macro to inject phenol red. The dye is observed circulating through the bloodstream and toward the heart. After completing the injection, the robot continues searching for the next larva to repeat the process.

Supplementary Movie 9. Semi-automatic injection of cancer cells into the duct of Cuvier (DoC). The robot automatically identifies larvae and DoC and navigates the needle toward the DoC. A user interface allows manual control for fine adjustments, including moving and rotating the needle, as well as performing the injection. The starting position and focus can be flexibly adjusted. Once the needle is correctly positioned, cancer cells are injected into the DoC.

Supplementary Movie 10. Reconstructed video after DoC injection. This video presents reconstructed images showing the distribution of the injected dye throughout the larva's blood circulation system.

Supplementary Movie 11. Automatic injection of cancer cells into the perivitelline space (PVS). The robot identifies a larva on the agarose gel plate and aligns the needle with the predetermined starting position for the PVS. The needle punctures the PVS at the predefined angle, and the user stops the injection when the desired cell volume has been delivered.

Supplementary Movie 12. Semi-automatic injection of cancer cells into the perivitelline space (PVS). The robot automatically identifies a larva and positions the needle at the starting point of the PVS. The user then uses a control interface to manually perform the injection.

Supplementary Movie 13. Reconstructed video after PVS injection. The video shows the successful delivery of cancer cells into the PVS.

Supplementary Movie 14. Automatic injection of phenol red into the hindbrain ventricle. After identifying a larva, the robot positions the needle near the hindbrain ventricle. The system detects when the needle reaches the ventricle's edge and executes the injection.

Supplementary Movie 15. Semi-automatic injection of glioma cells into the hindbrain ventricle. The robot positions the needle near the hindbrain ventricle, and the user uses a control interface to inject by selecting the “m” button.
